# Scientific diasporas and the advancement of science diplomacy: The InFEWS US-China program in the face of confrontational “*America First*” diplomacy

**DOI:** 10.3389/frma.2022.944333

**Published:** 2022-10-06

**Authors:** Julian Prieto, Christopher A. Scott

**Affiliations:** ^1^Department of Education Theory and Policy, College of Education, Pennsylvania State University, University Park, PA, United States; ^2^Department of Ecosystem Science and Management, Pennsylvania State University, University Park, PA, United States

**Keywords:** science diplomacy, diplomacy for science, scientific diaspora, WEF nexus, climate change

## Abstract

The challenges and consequences of climate change have brought together governments around the world to advance scientific knowledge and programmatic actions to develop mitigation strategies while promoting sustainable development. The United States and China—the countries with the highest science expenditures globally—have historically developed a range of joint international research collaborations. However, under the “*America First”* agenda put forth by the Trump Administration, bilateral diplomatic relations with China reached their highest confrontational peak. Under this scenario science diplomacy served as a catalyst to maintain scientific collaborations between both countries. In 2018, the US National Science Foundation and the China National Natural Science Foundation launched the InFEWS US-China program to promote collaborations to expand food, energy, and water nexus (FEW Nexus) research and applications. Over the past four years, 20 research projects have been awarded from the US side and 47 publications have been reported as research output. By carrying out a descriptive analysis of the InFEWS US-China research and scholarly outputs, we find evidence of the crucial role played by the Chinese scientific diaspora who led 65% of the projects awarded. We find that there is a generally good understanding of the interdependencies between FEW systems included in the project abstracts. However, in the InFEWS US-China scholarly outputs generated to date, there is a lack of usage of a clear FEW Nexus theoretical framework. Further research should address intentional policies that enhance the involvement of scientific diasporas in their home countries to better address climate, sustainability, and development challenges.

## Introduction

The US Presidential Administration of Donald Trump formally withdrew from the Paris Climate Agreement in June 2017, arguing that the commitments that it entailed were unfair to the American economy (Shear, 2017). The reasons used to justify this decision were based on the calculated cost of US$ 3 trillion in lost GDP and 6.5 million jobs in comparison to, it was claimed, more benevolent treatment received by emerging economies such as China and India (Matt McGrath, 2017). This decision threatened the collective efforts made by the international community to tackle the effects of climate change, mostly centered on strategies to reduce greenhouse gas emissions. In addition, based on his now trademark motto “*Make America Great Again”* (and “*America First”)*, Trump enacted a wide range of Neo-Nationalist policies with widespread impacts including those that greatly affected higher education institutions and research centers. Some actions included restrictive visa policies for students and professors as well as other anti-immigrant decrees beyond the education sector, anti-science rhetoric including the denial of the effects of climate change, and diminishing the attention and funding for programs to mitigate the severity of the COVID-19 pandemic (Douglas, 2021).

On the international diplomatic stage, relations between the US and China were at their highest confrontational peak under the Trump Administration (Beitelman, 2020). The “*America First”* Neo-Nationalist policies triggered a trade war against China by imposing, for example, tariff and non-tariff restrictions on Chinese imports. Furthermore, Trump accused China of theft of intellectual property and espionage through technology, telecommunications, and electronics companies in China, among many other actions (Boylan et al., 2021).

In the face of the diplomatic confrontation that these presidential decisions triggered, one science diplomacy mechanism stood out between the US and China in the field of climate change, specifically in relation to the food-energy-water nexus (FEW Nexus, also referred to as the WEF Nexus). The US National Science Foundation (NSF) partnered with the China National Natural Science Foundation (NSFC) in 2018 to launch the “Innovations at the Nexus of Food, Energy, and Water Systems (InFEWS: US-China)” program. An extension of NSF's broader InFEWS program that was launched in 2016, InFEWS US-China constituted the only sub-program that was carried out in partnership with a foreign government. Interestingly, NSF's domestic US InFEWS program has awarded in total 96 grants since 2016, while InFEWS US-China has awarded 20 from 2018 to 2022 as part of this total. This means that in just four years this bilateral program represents 21 percent of the total number of projects from the overarching strategy. It is important to understand why this program has been so effective in terms of awarding proposals and also how this program has served to advance research that addresses critical environmental sustainability challenges based on the FEW Nexus.

In this context, this paper will address three main research questions. First, what is the role played by the US-based Chinese scientific diaspora in promoting collaborations with scholars and institutions within China? Second, to what extent did the research projects approved by InFEWS US-China use the FEW Nexus concept, and did they adequately address the interdependencies among all three systems? Third, based on a review of all the publications reported to NSF by InFEWS: US-China projects as of April 2022, how did research teams use the FEW Nexus concept and how did their results contribute to the body of knowledge in this field?

In what follows, we present a brief history of the Sino-American research and scientific collaborations to elucidate how the InFEWS US-China extends a trajectory of binational diplomacy for science programs. Next, we discuss science diplomacy in light of the FEW Nexus conceptual framework, in particular, to assess whether integrated resource assessment and management might be more amenable to science diplomacy collaboration than, for example, a purely physical science approach. In the following section, we present the empirical research based on a descriptive analysis of data from the NSF website complemented by a SCOPUS dataset assessment using bibliometric information on the research output of all InFEWS US-China projects. This is followed by an analysis and discussion of findings, with specific attention to the research questions, as well as recognition of (1) the significant participation of Chinese scholars in the US as InFEWS US-China principal investigators, (2) the substantial partnerships developed with Nanjing Agricultural University, and (3) mixed success in understanding and usage of the FEW Nexus framework. Finally, in the conclusions, we synthesize the role of science diasporas in advancing science diplomacy, especially in the face of confrontational binational diplomacy.

## Brief history of Sino-American research and scientific collaborations

Chinese advanced scientific methods predated European and European-American advancement in many areas serving as a source of technological inspiration in the West (e.g., from pottery to textiles). Some authors, however, have documented formal Sino-American Science and Technology relations back to the Nineteenth century when US missionaries played the role of agents for transferring knowledge from science and engineering (Suttmeier, 2014). According to Suttmeier, in the first half of the twenteeth century, there was a fluid and growing scientific cooperation between both nations with an important role played by the cadre of US-trained Chinese scientists and engineers that took the lead in the knowledge transfer activities. 1949, however, represents a dramatic rupture of the scientific collaborations with the establishment of the People's Republic of China. Under Mao Zedong, official science and technology agreements were interrupted and scientific relations were relegated to non-official scientific relations that persisted between the US and Chinese scholars (Millwood, 2021). During the 1960s, American scholars created the Committee on Scholarly Communications with Mainland China (CSCMC) supported by the National Academy of Science, the American Council of Learned Societies (ACLS), and the Social Science Research Council (SSRC). The CSCMC was an independent and non-official initiative that facilitated the exchange of publications and meetings between the US and Chinese scholars in international conferences and aimed to maintain scientific collaborations despite difficult political relations (Smith, 1998).

It was under the Deng Xiaoping and Jimmy Carter governments in the 1970s that official science and technology cooperation was restored through the signature of the “US-China Inter-governmental Science and Technology Agreement”. Through this agreement, a Joint Commission on US-China Cooperation in Science and Technology was created and several programs and sub-agreements were put in place to foster higher research and innovation cooperation and most importantly opened the space for more Chinese graduate students in the US (Smith, 1998).

While on the political and diplomatic side, Sino-American relations have faced ups and downs since the 1980s (Niu, 2010), scientific cooperation has yielded significant results in various fields. In the last four decades cooperation achievements include a Remote Sensing Satellite Ground Station, the Beijing Electron-Positron Collider, the China Digital Seismograph Network, second-generation internet technology, as well as advancements in high-energy and nuclear physics, magnetic confinement fission, surface water hydrology, electric car, and fuel cell vehicle technology development, advanced reactor technology, and most recently, useful progress in agricultural S&T, clean energy, bio-medicine, wireless communication technology, and more (Suttmeier, 2014).

Scientific cooperation in a knowledge economy faces challenges in terms of disputes over intellectual property rights, patents, information security, export control restrictions, and trade barriers, among others (Stiglitz, 1999). In many cases, governmental policies centered on protecting national interests end up hindering and threatening scientific collaboration. However, it is precisely science diplomacy strategies and tools that nevertheless allow for scientific cooperation to advance and influence not only local and national policy, but also influence the international arena.

## Science diplomacy

Science Diplomacy is a growing field both in academia and in practice that focuses on the relationship between formal international relations and scientific cooperation. Although this term has been recently used officially by diplomats and scientists, examples of science diplomacy programs have been documented extensively since the Cold War (Turchetti, 2020). The difference between this concept and the independent scientist cooperation approach is the intention that governments may have when fostering programs that use scientific knowledge as the base of diplomatic relations and to promote national interest (The Royal Society, 2010).

There is not a universal consensus on the definition of science diplomacy. However, the initial attempt to define it was in 2009 by the Royal Society and the American Association for the Advancement of Science (AAAS) in the framework of a two-day meeting on science diplomacy. In their published report, three main dimensions of Science Diplomacy are established, namely, *science in diplomacy* (giving scientific advice for foreign policy decisions), *science for diplomacy* (relying on scientific cooperation to advance international relations purposes among countries), and, *diplomacy for science* (developing international programs to foster scientific cooperation) (The Royal Society, 2010).

Much research has been carried out on different scientific programs and strategies to advise on foreign policy (science in diplomacy). Historical studies have documented the long-standing scientific efforts and influence on international ocean policy (Robinson, 2020). Other studies have researched the evolution of global environmental programs led by scientists around the world (Rispoli and Olšákov, 2020). A contrasting example is the case of the Intergovernmental Panel on Climate Change that despite having a weak impact in terms of diplomatic decisions and governments commitment, it has been a positive international effort that provides sound scientific evidence for policies on climate change (Ruffini, 2018), in addition to significantly galvanizing global scientific consensus and popular opinion.

Likewise, the use of science in contexts of tense international relations has been documented extensively. Historical studies have shown the pivotal role that science collaborations played in the aftermath of World War II as a peace-building tool (Miller, 2006). The important role of scientific collaboration in avoiding a latent nuclear conflict during the Cold War has inspired many authors in analyzing science for diplomacy mechanisms (Barth, 2006; Turchetti, 2020).

Diplomacy for science, in contrast, has been a field of less research and attention (Linkov et al., 2016). Some studies have focused more on the role that science diplomats play to acquire information regarding the host nation's scientific priorities and the responsibility to foster research collaborations programs that support national interests (Linkov et al., 2014). Some initiatives that have resulted from diplomacy for science policies are the creation of binational science and innovation centers. These centers require the efforts and collaboration of universities, research institutes, think tanks, innovation organizations, and public institutions. The cases and best practices from Germany and Switzerland shed light on the positive impact of these efforts (Epping, 2020). Other types of analysis found are the impact assessment reports of international research collaboration programs. For example, the United Kingdom's Newton Fund program has produced several evaluation reports for different countries. For the China-UK program, the assessment was carried out through case studies documenting the results of research collaboration programs in precision agriculture, breast cancer innovative therapy, and climate science for service partnership (Department for Business, Energy, and Industrial Strategy, 2022).

The global and common environmental threats that the world faces underline the importance of science diplomacy. Particularly, the constraints, shortages, and scarcity of food, water, and energy as a consequence of climate change, were recognized at the Royal Society and AAAS conference in 2009 (The Royal Society, 2010). Indeed, some of the earliest programmatic development of “nexus” interlinkages between food and energy (Scott et al., 2015) were pioneered by the United Nations University, arguably a science for diplomacy institution of the international community. The Water, Energy, and Food nexus has become a field of special interest for science diplomacy programs and initiatives for many of the above-mentioned reasons. Our intent here is not to review the Nexus framework but to introduce those elements that are particularly salient to the science diplomacy focus of the present *Frontiers* special issue.

## The FEW nexus framework

Definitions and theoretical frameworks of the FEW Nexus are contested and this is a field in constant development. Insights on the coupled linkages between the three systems include not only the nexus assessment of resource quantification but also resource management and policy.

In a comprehensive review Albrecht et al. (2018) analyzed more than 245 papers that have been published under the FEW Nexus approach. However, they point out that much of the research carried out does not integrate appropriately the analysis of the interdependencies between the three systems. In addition, they identify that many studies are water-centered and carry out assessments of the interdependencies of just two systems. From the overall review, just 18 papers represent best practices in the implementation of interdisciplinary, participative, and mixed (qualitative and quantitative) research methods.

Some authors introduce a nexus framework centered on resource recovery as a fundamental biophysical expression to diminish the human footprint on planetary boundaries (Scott et al., 2015). This framework analyzes the interlinkages of the FEW Nexus on three planes, namely, biophysical resources, institutions, and security. Going further, some other authors focused on understanding the integration through three alternative perspectives, including incorporation, cross-linking, and assimilation (Al-Saidi and Elagib, 2017).

Modeling frameworks have been also introduced to assess FEW Nexus interlinkages. Some examples are the Multi-Scale Integrated Assessment of Societal Metabolism (MuSIASEM) which focuses its analysis on the metabolic patterns of socio-ecological systems counting for different hierarchical levels, scales, and dimensions of analysis (Giampietro and Mayumi, 2000; Pérez-Sánchez et al., 2019). A *FEW Nexus tool* has been introduced based on the analysis of the flows and interconnections between the three systems taking into account inputs and outputs of an integrated system allowing for modeling of different scenarios (Daher and Mohtar, 2015; Daher et al., 2017). Laspidou et al. (2020) introduced the *Nexus_SDM* as a systems dynamics tool with visualization to highlight WEF interlinkages.

The NSF considers the Nexus to be an example of its “10 Big Ideas” in its call for “Convergence Research.” As a result, we conjecture that the FEW Nexus is more amenable to science diplomacy collaboration than, for example, purely physical science programs.

## InFEWS US-China program

The US NSF InFEWS program was created in 2016 with the following three main goals: (a) support integrated experimental research toward creating a comprehensive food-energy-water sociotechnical systems model; (b) advance knowledge/technologies that foster safer, more secure, and more efficient use of resources within the food-energy-water nexus; and (c) support an integrated approach to building the next-generation INFEWS workforce. According to the NSF program webpage, 96 projects have received funding with a total allocated amount of US$ 167,569,869.

In 2018, a joint program with the Chinese NSFC was launched to promote collaborations between the US and Chinese scholars and researchers to advance in the Food, Energy, and Water Nexus.^1^ Specifically, InFEWS US-China called for proposals on the themes of (1) Quantitative and computational modeling of a Food, Energy, and Water (FEW) System, and (2) Innovative human and technological solutions to critical FEW systems problems. The program requested research proposals to be submitted by research teams in both countries to their corresponding agencies, namely, US scholars to the NSF and Chinese scholars to the NSFC. The funding provided to winning proposals by each agency was on average US$500,000 for projects to be implemented for up to 4 years. The program terms of reference established the condition to include the participation of researchers from at least one US institution and at least one institution in China.

Between 2018 and 2021 20 projects totaling $8 million were granted by the NSF to US research teams. Although public information is not available, this means that the same number of projects must have received funding on the China side. According to publicly available data on the NSF webpage, the winning teams on the US side have reported the publication of 47 research papers funded under the INFEWS US-China program. This information was not accessible from the NSFC webpage.

## Data and methods

This paper carries out a descriptive analysis on three levels. The first focuses on assessing the research collaborations patterns and the role of the Chinese scientific diaspora in the InFEWS US-China program as an example of the impact of a diplomacy for science mechanism. The second centers on determining if the research proposals awarded by the NSF show a clear understanding of the FEW Nexus by incorporating the interlinkages of the three systems in the abstracts of the winning projects. Finally, a review of all the research papers reported to the NSF under the program is carried out focusing on the use of the FEW Nexus concept.

A first dataset was downloaded from the NSF webpage with the report of all the grants awarded for the InFEWS US-China program. This dataset was filtered to identify all the grants awarded under the US-China program. Next, each project was searched on the web under its award code to access the summary report of each project on the NSF webpage. This provided information about the leading institution in the US, the partner institution in China, the principal investigator (PI), the abstract of the full proposal, and links to all the self-reported publications from each project. This dataset was complemented by searching on the web for each PI to identify the institution where they obtained their bachelor's degree under the reasoning that if authors studied as undergraduates in a Chinese university, they are likely Chinese. In addition, information about their gender and institutions of graduation from all levels were codified. Lastly, each project was classified on either of the two program themes (1. Computational modeling or 2. Technology innovation) by extracting the information from the abstracts.

The second level of data analysis was carried out using SCOPUS to search for and create a list of each reported publication. Of the 47 publications reported, 7 papers were not found in SCOPUS. All the bibliometric information, including title, authors' names, institutional affiliations, keywords by authors, abstract, citations index, and funding sources were downloaded to an excel file. The operational analysis of this data is shown in Table 1.

**Table 1:** Operationalization of data.

**Operationalization method**		
**Source of data**	**Unit of analysis**	**Analysis**
NSF proposal summary information	Principal investigator's (PI's) undergraduate institution	Does the PI have a Chinese background? Are the Chinese researchers collaborating with the institutions they graduated from?
	Abstract analysis	To what theme does the project respond? Does the project abstract establish a clear interlinkage of the three FEW Nexus systems?
SCOPUS publication bibliometrics	No. of co-authors	Patterns of collaboration
	Is the PI of the NSF project co-author of the publications and in what authorship position	Is the research project led exclusively by the PI or are there emerging scholars in the field?
	Is the publication co-authored with scholars from the Chinese partner institutions	is there evidence of a real binational research collaboration?

Consequently, the sample covered in this review includes all the winning proposals awarded by NSF as well as the corresponding self-reported publications associated with each project. A summary of the winning proposals identifying the US institutions and their corresponding Chinese partner institutions is shown in Table 2. Information is reported also on the amount granted in dollars and the papers reported to NSF as a result of the grant.

**Table 2:** InFEWS US-China projects funded by NSF (information extracted through the NSF webpage).

**US Institution**	**Chinese Partner Institution**	**Awarded Amount (US$)**	**Publications**
Purdue University	Center for Energy and Environmental Policy, State Key Laboratory, and Center for Chinese Agricultural Policy	$499,341.00	2
University of Illinois at Urbana-Champaign	China Agricultural University	$500,000.00	5
Louisiana State University	Dalian Ocean University	$291,788.00	1
Texas A&M University Corpus Christi	Dalian Ocean University	$208,087.00	0
Vanderbilt University	Harbin Institute of Technology	$177,914.00	0
Columbia University	Harbin Institute of Technology	$364,710.00	1
University of Missouri-Columbia	Jiangnan University in China	$500,000.00	0
University of Utah	Nanjing Agricultural University	$149,845.00	1
University of South Carolina at Columbia	Nanjing Agricultural University	$199,942.00	0
California State University-Fresno Foundation	Nanjing Agricultural University	$149,818.00	0
Virginia Polytechnic Institute and State University	Nanjing Agricultural University, and Wuhan University of Technology in China	$500,000.00	3
University of Tennessee Knoxville	Nanjing Agriculture University	$500,000.00	0
University of Maryland, College Park	Nanjing Hydraulic Research Institute, Chinese Academy of Sciences, and the Northwest Agriculture and Forestry University.	$500,000.00	6
Lehigh University	Not Reported	$499,891.00	3
Auburn University	Not Reported	$500,000.00	13
University of Maryland Center	Not Reported	$500,000.00	2
North Carolina State University	Not Reported	$500,000.00	0
University of Delaware	Tianjin University	$500,000.00	7
West Virginia University Research Corporation	Zhejiang Sci-tech University	$494,888.00	1
New Jersey Institute of Technology	Zhejiang University	$500,000.00	2
Total		**$8,036,224.00**	**47**

## Analysis and discussion of findings

### Collaboration patterns

Chinese diaspora organizations and its collaborations patterns with scholars in their homeland have been portrayed as a best practice. Some reasons for this success rely on the high number of Chinese scholars that leave overseas (over a million), their capacity to create more than 200 diaspora organizations, and the intentional policies at the federal and provincial levels (Meyer, 2011). The social capital theory has been used to explain the success of this fluid scientific collaboration stressing the importance of language and cultural understanding that facilitates and enables successful scientific collaborations (Biao, 2005). In the case of the InFEWS US-China program, 65 percent of the PI's (13 in total) did their bachelor's degrees in China (see Table 3). Six projects led by these Chinese scholars have published with Chinese co-authors affiliated with institutions in China. This represents an initial understanding of how the social capital and networks of Chinese scholars function under the InFEWS diplomacy for science mechanism.

**Table 3:** Nationality of principal investigators.

**China**	**USA**	**Taiwan**	**India**	**Singapore**
13	4	1	1	1

**Figure 1:**
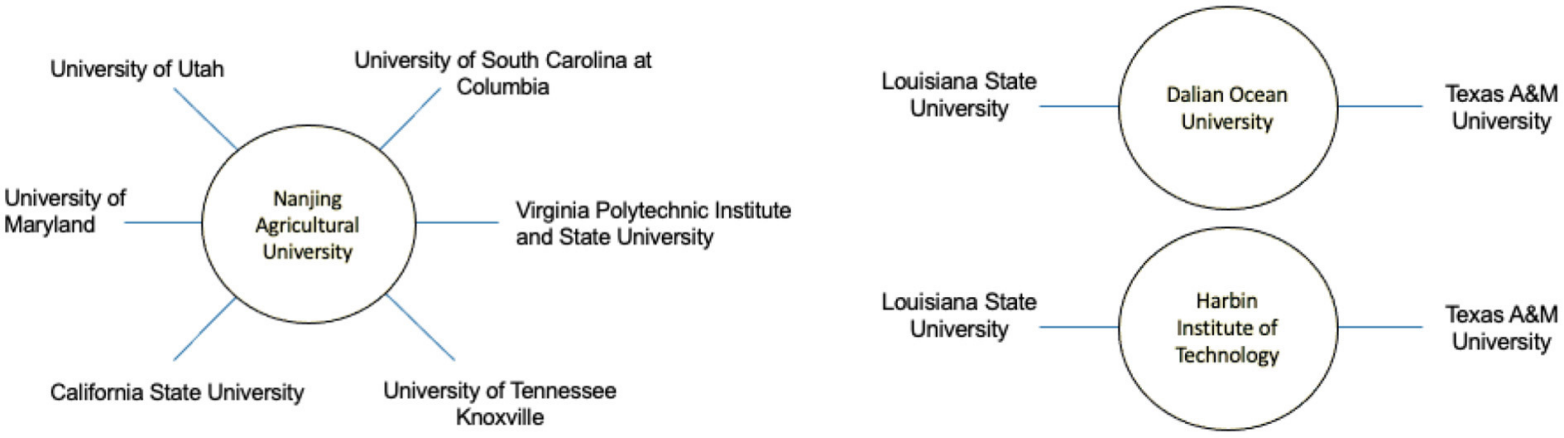
InFEWS US-China collaboration networks of selected Chinese universities.

As shown in Figure 1, Nanjing Agricultural University stands out as the main Chinese partner institution collaborating with 6 out of the 20 winning proposals. This university is recognized worldwide as a leading research institution in agricultural, plant, animal, and environmental science according to the World University Ranking^2^ and is placed number 135 in the Best Global Universities.^3^ Its internationalization strategy and research network include over 150 partnerships and agreements around the globe and has been taking part in large international research projects with the European Union, the US, and United Nations Programs reported by World University Ranking. The universities of Dalian and Harbin Institute of Technology each have two partnerships under the InFEWS program. No Chinese institution partner was found for four of the projects listed.

Specifically analyzing the bibliometric information from all the 40 publications under review the average number of co-authors is 8.5 with a minimum number of authors of 2 and a maximum of 57. Thirty-one of the 40 publications listed the PI of the NSF project as co-author, but not necessarily as first author (which was the case for just five papers). This may be evidence that PI's are promoting the engagement of young scholars. Finally, 21 publications have scholars affiliated with institutions in both countries. However, just 10 of these 40 publications are co-authored with scholars from the official Chinese partner institution.

### FEW nexus assessment in NSF InFEWS US-China proposal abstracts

The analysis carried out in this section is based on the abstracts of the 11 projects that have reported publications and were found in SCOPUS. Of the total, six proposals respond to computational modeling challenges, three tackle technology innovations and three address both themes.

All the proposals from the computational theme establish a clear interlinkage between the three FEW systems. Every abstract mentions individually each of the systems of the FEW and establishes a clear relationship between them. The computational models proposed in some cases rely on models developed for each of the systems but propose bridges between these models (Award No. 1804560 and 1805808). Two projects propose to carry out comparative FEW Nexus analysis on specific locations in both countries (Award No. 1903722: Mississippi River Basin and Yellow River Basin, 1903249: Yellow River Basin). An innovative study integrates wave energy-based seawater desalinization systems, sustainable reclamation of saline-sodic alkaline soils, and a nexus of ocean energy, freshwater, and coastal agriculture (Award No. 1903627).

In contrast, none of the three projects presented under the technological innovation theme demonstrates a clear understanding of the interdependencies of the FEW Nexus. Award No. 1803200 focused on electrocatalyst for CO_2_ conversion using electrochemical processes. They do test some renewable energies in the process but the link between water and food is not clear. Award No. 1903597 proposes a biological active filtration system to purify water resulting from rice crops. The nexus between water and food is clear in this case, however, energy is not incorporated. Lastly, Award No. 1903705 focuses on its analysis of integrated treatment for source-separated urine. Although they point out the importance of the resulting finding for nitrogen and phosphorous recovery that may be essential for agriculture production, they do not highlight this connection, nor do they establish a relationship with the energy system.

The remaining two projects addressing both themes show a clear understanding and evidence of the interdependencies of the FEW Nexus in their proposals. Award 1804453 is focused on modeling and developing technology to carry out hydrothermal liquefaction techniques for the conversion of wet biowaste and algae into biocrude. Award 1804453 models the impact on how petroleum products impact the development and growth of oysters due to the changing quality of water.

This analysis shows that on the computational modeling proposals there is a clear understanding of the FEW interdependencies, although few if any projects addressed the policy implications of their work either in site-specific resource management terms or, for the interests of this paper, for science diplomacy. None of this was the case for the technological innovations projects that may be focused on tackling very particular issues that do not manage span the FEW Nexus interlinkages.

### Review of the research output reported to NSF

This section will follow the same structure as the previous one by considering InFEWS US-China program themes. When filtering by keywords the computational modeling projects none of the 25 publications includes the word “nexus”, four include the word “water”, three the word “energy”, and one the word “food”. If trying to look for alternative ways to search for a coupled approach five papers are found with the word “climate”. From the water-centered publications, one establishes a water-energy nexus by analyzing the effects of climate change on the hydroecological conditions and natural hazard risk (Yang et al., 2019). Another publication establishes a water-food nexus by analyzing the effects of conservation tillage used in corn-soybean on crop water productivity (Huang et al., 2021). Publications from this research project (Auburn University) are related either to nitrous oxide quantification (Tian et al., 2020a,b; Yao et al., 2020; Bian et al., 2021) or to evapotranspiration (Pan et al., 2020). Despite their lack of direct usage of a nexus definition, their findings contribute solidly to the FEW Nexus field.

The energy papers are mostly centered in their own unique field (Ogunrinde et al., 2018; Mi et al., 2020), with exception of one paper that analyzes the moist heat stress on farmers' productivity (Buzan and Huber, 2020). The University of Maryland makes significant contributions through six publications focused on precipitation and climate models. Although they do not mention the nexus approach, their findings are pivotal to assessing models that incorporate the effects of climate change in accounting for changing patterns of precipitation for agriculture and hydroelectric energy (Sun and Liang, 2020a,b; Sun et al., 2020; Li et al., 2021). It is important to highlight that almost all publications from this are co-authored by researchers from their partner, Nanjing Agricultural University, which may result from a project governance policy developed by the U Maryland and Nanjing U team.

On the technological innovation theme, there are nine publications in total but just one publication is co-authored by scholars from both countries. The partnership between the University of Delaware and Tianjin University contributes six publications reporting mainly on their advancement in the electrochemical conversion of CO_2_ methods and technologies (Jouny et al., 2019; Luc et al., 2019; Ko et al., 2020; Xia et al., 2020). The remaining publications introduce the technology of using biochar to purify water (Liu et al., 2021) and an isothermal membrane distillation with an acidic collector for the recovery of ammonia from urine (McCartney et al., 2020).

Finally, of the projects that tackle both themes, one is led by Louisiana State University with a paper that analyzes the Mississippi River discharge impact on the Barataria estuary salinity and its effects on marine life (Ou et al., 2020). The other one is carried out between the University of Illinois and China Agricultural University with all binational publications, again possibly reflecting specific project governance agreements between the PIs. The five publications from this partnership are focused on progressing in refining the techniques for biocrude production using hydrothermal liquefaction methods. This project shows different experiments that they have done using different types of livestock and techniques (Stablein et al., 2020; Watson et al., 2021a,b). One of the most interesting projects carried out is the one where they use food waste from a university campus and combine it with wastewater to produce biocrude (Aierzhati et al., 2021). Their research is pushing the boundaries not only in technological innovation but also in quantifying its economic viability (Watson et al., 2020) and at the same time expanding the boundaries of FEW Nexus research and applications.

This study has several limitations that we consider to be avenues for future research. First, the lack of information on NSFC-approved projects, either in relation to resources allocated, partnerships, institutions, or PI's, impedes a complete picture of InFEWS US-China. In addition, as none of the projects has completed implementation such that the final outcomes are not yet available, our review covers work in progress and should not be taken as a comprehensive analysis of the potential contributions of the program to advancing the FEW Nexus framework, given that many of the projects may be in the middle of their implementation process. Additionally, in terms of methods, the collaboration patterns can be systematically and more reliably carried out using network analysis tools (e.g., see Dennis and Grady, 2022) that would allow an analysis not only of the project PI's but also a full network analysis of the co-authors to identify previous patterns of collaboration that may explain the degree of success of certain institutional partnerships. Finally, an interactive survey of InFEWS US-China project team members, particularly concerning their assessment of science diplomacy objectives, would provide unique insights to both expand and solidify science diplomacy as well as to expand global understanding of the FEW Nexus.

## Conclusion

The InFEWS US-China program is a clear example of diplomacy for science. The collaboration patterns show that scientific advancement in the field of climate change requires collective efforts indicated by a high average number of co-authors per paper. These collaborations in many cases are stronger when incorporating different perspectives, contexts, and cultural backgrounds. Therefore, promoting and enhancing major international collaborations through diplomacy for science programs is a needed strategy that should be incorporated within all countries' international relations policies. Nanjing Agricultural University emerged as the leading partner institution in China. This paper does not show evidence of a specific reason for this to occur, however, the long-standing international relations by this institution may explain a global engagement culture not common in many Chinese universities.

In terms of the FEW Nexus advancement, it is clear that the framework is well incorporated within the NSF proposals by establishing in most cases clear interlinkages among the three systems. However, the research output does not support a clear appropriation of the concept by the research teams. Although much of the research findings may make significant contributions to FEW Nexus analysis, many of the research outputs do not incorporate the FEW Nexus within its explanatory frameworks or conclusions. Finally, few if any projects explicitly considered the broader policy implications of their research (for FEW management, climate change adaptation or mitigation, sustainable development, or human security).

Despite the Trump Administration publicly promoting anti-climate change policies and carrying out confrontational diplomatic relations against China, the InFEWS US-China program maintained and extended scientific collaborations between US-based and Chinese scholars in the fields of climate change and sustainable resource management. As demonstrated above, there is an important role played by the Chinese scientific diaspora leading 65 percent of the winning InFEWS US-China proposals. The social capital theory has been used to explain the success of these dynamic scientific collaborations stressing the importance of language and cultural understanding that facilitates and enables successful scientific collaborations (Biao, 2005). Although we were only indirectly able to address the role or willingness of the Chinese scientific diaspora in the US in moderating the confrontational “*America First”* diplomatic context in which InFEWS research was initiated, it is evident that multiple binational teams have conducted, and continue to develop successful science policy research. Furthermore, the strength and cohesion of the Chinese scientific diaspora and the policies promoted by the Chinese government may serve as inspirations to diaspora networks from other countries to strategically contribute to their homeland while living abroad (Shin and Moon, 2018).

This program shows evidence of the important role played by the Chinese scientific diasporas in developing research collaborations with their home countries. This may serve as a good example of the transforming concept of Brain Drain into Brain Circulation (Fangmeng, 2016). Governments may use this case as source of inspiration to design policies that incorporate not only incentives for high skilled scientist to go back to their home countries, but also develop incentives for those scientists that want to stay abroad and build networks with their local higher education system.

## Author contributions

JP and CS contributed to conception and design of the study. JP created the NSF InFEWS database and wrote the first draft of the manuscript. CS revised and made improvements on the first draft. Both authors contributed to manuscript revision, read, and approved the submitted version.

## Conflict of interest

The authors declare that the research was conducted in the absence of any commercial or financial relationships that could be construed as a potential conflict of interest.

## Publisher's note

All claims expressed in this article are solely those of the authors and do not necessarily represent those of their affiliated organizations, or those of the publisher, the editors and the reviewers. Any product that may be evaluated in this article, or claim that may be made by its manufacturer, is not guaranteed or endorsed by the publisher.

## Footnotes

^1^Links to NSF InFEWS US-China calls: 2018: https://www.nsf.gov/pubs/2018/nsf18096/nsf18096.jsp. 2019: https://www.nsf.gov/pubs/2020/nsf20019/nsf20019.jsp. 2020: https://www.nano.gov/node/2890. 2021: https://www.nsf.gov/pubs/2021/nsf21103/nsf21103.jsp?WT.mc_id=USNSF_25&WT.mc_ev=click.

^2^ https://www.timeshighereducation.com/world-university-rankings/nanjing-agricultural-university

^3^ https://www.usnews.com/education/best-global-universities/nanjing-university-503849#summary
